# Supplementary material for: Engaging Community Health Workers (CHWs) in Africa: Lessons from the Canadian Red Cross supported programs
Source: PLOS Glob Public Health. 2024 Jan 18;4(1):e0002799. doi: 10.1371/journal.pgph.0002799 (PMC10796059; doi:10.1371/journal.pgph.0002799)
Supplement: S1 Fig — (PDF) [file pgph.0002799.s001.pdf]

Supplement 1 Figure. Barriers and Facilitators of CRC supported CHW projects

Barriers and Facilitators to Implementing CHWs

Barriers

Contextual Factors

- Political:
  - International sanctions and border closures impeded access to project sites (Mali, 2016-2021)
  - Post-electoral crisis resulted in delay of activities (CAR, 2020-2023)
  - Conflict areas resulted in fear and challenges accessing facilities (South Sudan, 2019-2022)
  - Security situation hampered activities (Kenya, 2012-2015)
- Climate:
  - Flooding and rainy season impeded access to project sites (South Sudan, 2011-2012; Guinea 2014-2018; Kenya 2012-2015)
- Pandemics and Outbreaks:
  - COVID-19 Pandemic delayed start project (CAR, 2020-2023)
  - Lockdowns delayed implementation of activities (Somaliland, 2021-2022)
- Infrastructure:
  - Target villages were remote, difficult to access and had high incidence of malaria (Kenya, 2012-2015)
  - Geographic, economic, and social barriers caused limited access to health services in addition to Ebola Outbreak (Liberia 2012-2015)

Organizational Structure

- Resources:
  - Limited funding and resources to secure adequate medication stock for integrated community care management (iCCM) (Liberia, 2012-2015)
  - Limited budget to purchase pharmaceutical kits for CHW (Mali, 2011-2015)
  - Instability in availability of medical equipment, which causes major risk to sustainability of skill transfer (Guinea, 2014-2018)
  - Poor organizational structure, mutually acceptable operational modality and inefficient project management impeded implementation (Guinea, 2014-2018; Liberia, 2014-2018)
- Human Health Resources:
  - Low literacy levels led to the poor organization of record keeping and reporting (Liberia, 2014-2018; Liberia, 2012-2015; Kenya, 2012-2015)
  - High staff turnover due to competing household demands (Liberia 2012-2015)
  - Inadequacy and low motivation among workers (Guinea, 2014-2018)
  - Transportation and district coverage was challenging given the long distance (Guinea, 2014-2018)

Training of CHWs

- Literacy and Developmental Levels
  - Low literacy levels among volunteers (Liberia, 2014-2018; Liberia, 2012-2015; Kenya, 2012-2015)
  - Poor record keeping and reporting (Liberia ,2014-2018; Liberia, 2012-2015; Kenya, 2012-2015)

Facilitators

Contextual Factors

- Open, timely, communication with MoH activities and engaging with community leaders (Mali, 2011-2015)
- Local volunteers/actors can help with access when security issue for expatriate staff; partner with local actors to leverage resources (i.e., example of UNICEF) (Mali, 2011-2015)
- Mitigate risks of migration by providing local authorities with early warnings of insecurity based on surveillance system to detect changes in communities (Kenya, 2012-2015)
- Regular reporting process and monthly updates, challenges and lessons learned between partners to facilitate integrated programming to changing environments (Kenya 2012-2015)
- Field presence of partner national societies (i.e., CRC) for technical support and training of staff and volunteers integral to managing project implementation (Somaliland, 2021-2022).
- Working with communities and relevant Ministries to scale-up delivery of basic health services to poor people living in rural areas (Kenya, 2012-2015).
- Agility and flexibility in project implementation and planning despite unforeseen risks (i.e., no in-country partner delegate, pivoting to provide MNCH services while also helping with Ebola outbreak) (Liberia, 2012-2015)

Organizational Structure

- Ensuring proper stock monitoring of medications to allow for timely access to pharmaceuticals (Liberia, 2012-2015).
- Negotiations with UNICEF who agreed to provide all components of the kits.
- Regular joint supervision visits not only contributed to motivation of CHWs, but they also ensured proper stock monitoring of medication for timely access to pharmaceuticals (Mali, 2011-2015).
- Regular coordination meetings were organised by the project national, regional and district teams in partnership with the Ministry of Health. This also included the MNCH task force and national groups. This encouraged open and timely communication and cooperation on at the district and national levels, and helped integrate supervision and monitoring, share lessons learned etc. (Mali, 2011-2015)
- Collaborative meetings with the community leaders, the MoH, CRM local chapters and national Heads Quarters to develop the project sustainability plan. This plan defined the role and responsibility of the different stakeholders (Mali, 2011-2015)
- Continuous supportive supervision, monitoring, and evaluation of the project, which ensured effective implementation and accountability (Liberia, 2012-2015)
- Regular joint supervision (i.e., CHW supervisor) visits contributed to motivating CHWs, increased CHW capacities and visibility, reinforcing learning and practical application (Guinea, 2014-2018; Mali, 2011-2015)
- Coaching approach used resulted in more acceptance and created an effective learning space for staff (Guinea, 2014-2018)
- Implementing a supervision book where observations, recommendation and action plans for each visit are recorded for follow up and monitoring purposes, and encourage/motivate staff to abide the action plans (Guinea, 2014-2018)
- Provision of incentives for volunteers, such as bicycles and mobile phones, to enhance their motivation and performance (Liberia, 2012-2015)
- NSs provided volunteers for the project through their network of volunteers (Mali 2016-2021, Kenya,2012-2015)
- Community meetings resulted in communities agreeing to participate and the training of volunteers. 154 volunteers were identified following these visits (CAR, 2020-2023)
- MoH provided public health officers from local health facilities (Kenya, 2012-2015)

Training of CHWs

- Training and capacity building of volunteers, which enhanced their knowledge and skills to provide quality health services. (Liberia, 2012-2015)
- Regular joint supervision (i.e., CHW supervisor) visits contributed to motivating CHWs, increased CHW capacities and visibility, reinforcing learning and practical application (Guinea, 2014-2018; Mali, 2011-2015)
- Coaching approach used resulted in more acceptance and created an effective learning space for staff (Guinea, 2014-2018)
- Starting each visit with a roundtable where recommendations and action plan of the previous visit is reviewed help keep on track, "responsibilise"/empower staff and improve implementation of recommendations by the staff. Locally-designed & developed toolbox used by volunteers for community sensitization increases beneficiaries' interest & attention during the sessions, hence messages retention

Barriers and Facilitators to Implementing CHWs

Barriers

Participatory Approaches

- Engagement:
  - Challenges to mobilize communities (Liberia 2012-2015)
  - Mistrust within medical institutions developed during Ebola outbreak, which affected prevention and identification of Ebola virus (Guinea 2014-2018)
  - Home visits to women who gave birth traditionally not done by men when there are no female CHWs this presents a barrier to access (Mali, 2011-2015)

Integration of CHWs and Programs into Local Health Infrastructure

- Infrastructure
  - High staff turnover due to competing household, business, and family demands (Liberia 2012-2015)
  - Weak health care infrastructure makes it difficult to implement health programs (Liberia 2012-2015)

Facilitators

Participatory Approaches

- Strong support from local authorities, traditional leaders, and communities and used a community-driven approach that empowered communities to take ownership of the project (Liberia, 2012-2015).
- Partnership and collaboration with the Ministry of Health and Social Welfare, and other stakeholders (Liberia, 2012-2015).
- In South Sudan, ownership from the communities was very high. Community members were involved from the very onset of the project, facilitating commitment and accountability. Activities were monitored and assessed through regular meetings at the facility, facilitating strong will from the community to run the facility in the long run (South Sudan, 2019-2022)
- To ensure community ownership of the project's interventions and to initiate the process of identifying community volunteers, field visits were organised to meet with the various communities. These meetings brought together in each village visited, in addition to community members, village chiefs, women's leaders, youth presidents, traditional birth attendants, traditional healers, religious leaders and community judges. (CAR, 2020-2023)
- Strong support from local authorities, traditional leaders, and communities
- Use of a community-driven approach that empowered communities to take ownership of the project.
- Partnership and collaboration with the Ministry of Health and Social Welfare, and other stakeholders.
- Sensitization sessions lead by key community influencers (e.g., imams in mosques) with support from RCV increases coverage, reduces resistance, and increases practice of recommended behaviours by community members (Guinea, 2014-2018).
- Male CHWs were accompanied by female Mali Red Cross volunteers to facilitate SRHR work with women/girls (Mali, 2011-2015).

Integration of CHWs and Programs into Local Health Infrastructure

- Critical for plans to be integral/ integrated with MoH Plans (Guinea)
- The CRC and CRM established six districts coordination teams which bring together the MoH and CRM. These teams worked closely with the MoH staff at each district to ensure the effectiveness and relevance of interventions, to facilitate learning and ensure coherence with MoH strategic objectives (Mali, 2011-2015)
- Enhanced community ownership and sustainability through coordination with the MoH and local partners. The community level involved introducing the project to leaders and community members to ensure their participation. Handover sessions and events were organized at various levels, including reviewing trainings, providing certificates, and developing an emergency preparedness plan. Despite challenges, the stable and reliable field team contributed to the project's success, working independently during a period of instability. (Liberia, 2014-2018)
