# Supplementary material for: Engaging Community Health Workers (CHWs) in Africa: Lessons from the Canadian Red Cross supported programs
Source: PLOS Glob Public Health. 2024 Jan 18;4(1):e0002799. doi: 10.1371/journal.pgph.0002799 (PMC10796059; doi:10.1371/journal.pgph.0002799)
Supplement: Attachment — Submitted filename: Response to Reviewers_02-10-2023.docx [file pgph.0002799.s003.docx]

**October 20, 2023**

**Response to Editor & Reviewers Comments**

Manuscript PGPH-D-23-01553

***Engaging Community Health Workers (CHWs) in Africa: lessons from the Canadian Red Cross supported programs***

**Editor Comment**

Journal Requirements: Some material included in your submission may be copyrighted. According to PLOS’s copyright policy, authors who use figures or other material (e.g., graphics, clipart, maps) from another author or copyright holder must demonstrate or obtain permission to publish this material under the Creative Commons Attribution 4.0 International (CC BY 4.0) License used by PLOS journals. Please closely review the details of PLOS’s copyright requirements here: PLOS Licenses and Copyright. If you need to request permissions from a copyright holder, you may use PLOS's Copyright Content Permission form. Figure 1: please (a) provide a direct link to the base layer of the map (i.e., the country or region border shape) and ensure this is also included in the figure legend; and (b) provide a link to the terms of use / license information for the base layer image or shapefile. We cannot publish proprietary or copyrighted maps (e.g. Google Maps, Mapquest) and the terms of use for your map base layer must be compatible with our CC-BY 4.0 license.

RESPONSE: I have completed the revision of this article and I had one question about the Copyrights to the map. I used VISME (<https://my.visme.co/editor/WGoveEtpaldqVWJ2b1c2UGtCY25YQT09OjrAIv3ZqSaBFf9krp8d5SXt/data?template=ekRhYXExOUdVd0VzWW1Sanc0cUxFUT09Ojp40EtgciDSJebqFspeT06S>) to create the map. This is clearly marked on the map in the bottom right-hand side of the map.

Here's their VISME Terms and Conditions of Use - <https://www.visme.co/terms_conditions/>

This explanation was sent into PLOS Global Public Health and I received a positive response to move forward with the submission (communication with Ms Shaira Joy Petancio; communication also attached to submission). Happy to make changes accordingly.

**Reviewer #1**

Dear Authors, I want to commend you on the extensive work you have undertaken to examine Community Health Worker (CHW) programs supported by the Canadian Red Cross (CRC) in Africa. Your research is highly relevant, especially considering the critical role that CHWs play in healthcare delivery in low-resource and humanitarian settings. The manuscript does an excellent job of outlining the methodology and the breadth of projects considered. The use of the READ approach for document analysis adds a robust framework to your study. Furthermore, your findings contribute valuable insights into the facilitators and barriers affecting CHW programs, filling an essential gap in the literature. However, there are some areas where the manuscript could be improved for clarity, depth, and impact:

RESPONSE: Thank you very much for your positive words regarding our manuscript and your very welcome suggestions for improvement.

Abstract

- Please consider including the number of reports that were analysed in your document analysis. This would offer readers an idea of the study’s scope and the weight of the evidence presented.

RESPONSE: Thank you, agreed and we have revised accordingly (line 39)

- Another area that could make a stronger impact is the specificity of your findings. The abstract offers a broad summary, but highlighting one or two key, concrete findings could draw more attention and add more value to the research.
- RESPONSE: Thank you, agreed and we have revised accordingly (lines 44-53).
- Regarding your study objective, the aim to explore "key findings and key lessons learned" from CRC-supported CHW programs is clear but could benefit from greater specificity. What makes a particular finding key? You can be clear on whether you looking at health outcomes, program sustainability, cost-effectiveness, or some other measure of success? Providing a more targeted focus could add rigor to your study and make the objective more transparent to the reader.
- RESPONSE: Thank you, agreed and we have revised accordingly (lines 34-35).

Introduction

- Line 87-92 : the authors transition from discussing the role of CHWs in humanitarian crises to specific activities in Africa. While some regions in Africa do experience such crises, the continent is diverse, and not all areas are affected in the same way. Specifying which countries or regions in Africa you are referring to could add more nuance and accuracy to your discussion.

RESPONSE: Thank you, agreed and we have revised the section accordingly. The list of countries in the region of Africa has been clarified (lines 97-103).

- Line 100-103: the authors argue about "important knowledge gaps" regarding the implementation of CHW programs in humanitarian, low-resource, and conflict settings. While you mention that "important knowledge gaps remain," the introduction does not delve deeper into what these gaps specifically entail or cite existing research that has attempted to address them. Given that your study aims to contribute to filling these gaps, a more comprehensive review of current literature would significantly strengthen your introduction. For example, a study by Miller et al. (2020) reviewed the evidence base on CHWs in humanitarian settings and highlighted key challenges and facilitators that could be considered when implementing CHW programs in such contexts. Additionally, a systematic review by Werner et al. (2023) provides valuable insights into the effectiveness of CHW-delivered healthcare in post-conflict settings. Integrating the findings from these systematic reviews into your introduction could offer readers a richer context and better understanding of the specific knowledge gaps your study aims to fill. This would not only solidify the rationale for your research but also potentially strengthen its impact and relevance.
  - Miller NP, Ardestani FB, Dini HS, Shafique F, Zunong N. Community health workers in humanitarian settings: Scoping review. J Glob Health. 2020 Dec;10(2):020602. doi: 10.7189/jogh.10.020602. PMID: 33312508; PMCID: PMC7719274
  - Werner K, Kak M, Herbst CH, Lin TK. The role of community health worker-based care in post-conflict settings: a systematic review. Health Policy Plan. 2023 Feb 13;38(2):261-274. doi: 10.1093/heapol/czac072. PMID: 36124928; PMCID: PMC9923383.

RESPONSE: Thank you very much for your suggestions and supporting literature to enhance our introduction. We agree and have revised accordingly to include these reviews (lines: 112-128)

Methods

The authors did well in providing a comprehensive outline of how the analysed the retrieved documents. The READ steps used is well articulated. The authors may consider the comments below as areas that could be improved:

- The methods section would benefit from the authors stating the number of documents that were analysed. This will give readers an idea of the scale and scope of the study.

RESPONSE: Thank you, agreed and we have revised the section accordingly to indicate the number of project documents that were anlayzed (lines 216-217)

- It is a good practice to note that each project was extracted by two team members. The authors need to include a statement on how discrepancies between them were resolved. Was it through discussion? Or a third reviewer?
- RESPONSE: Thank you, agreed and we have revised the section accordingly to indicate how discrepancies were resolved (lines 229-231)
- While ethics approval is mentioned, the manuscript could expand on any additional ethical considerations, especially given the sensitive nature of humanitarian settings. The authors mentioned keeping the data in a secured Team’s Channel. I think this ensured confidentiality of the data. Were there other measures?
- RESPONSE: Thank you for your question. To clarify, the reports are housed in a secure data repository at CRC that requires clearance to access. Raw data with identifiers and sensitive information that were analyzed and lead to the reports (which we extracted) were not used. We used aggregate report data with no personal identifiers and reported aggregate numbers and key themes and we did not use raw data that named CHWs who participated in the evaluations of the interventions. Most of the data anlayzed were quarterly and final reports submitted by country officers working on the project and outlining lessons learned in the field throughout implementation.

Findings

- Line 268 - Your manuscript mentions 'Data extraction of the studies' but it seems the primary data source are reports supported by CRC. It would be more accurate to consistently refer to these as 'reports' to avoid any confusion about the type of data being analyze.

RESPONSE: Thank you, agreed and we have revised the section accordingly (line:295)

- **Line 224- 226:** the statement that the projects reached 'several countries across Africa' could be clarified to be more informative by specifying the exact number of countries involved in the projects. I would also like to see a clearer communication of the geographical limitations of the project’s implementation.
- RESPONSE: Thank you, agreed and we have revised the section accordingly, we specify the number of countries and the sentence proceeding outlines the details of the 8 country specific and 1 multi-country project (line: 260).
- Your presentation of the data in table 1 is quite clear. I noticed that the multi-country projects are grouped together in the table. Could you clarify the reason for this approach? Was it possible to include subtotals for these multi-country projects? This would offer a more consolidated view of the impact across individual countries involved in these multi-country initiatives.
- RESPONSE: Thank you for your question. The project was a Canadian International Development Agency (CIDA) funded project which began in 2007 where CRC would implement the Integrated Child Survival program in the 5 countries, it was a multi-country initiative and we have access to the multi-country project outcomes and not the individual countries involved. It is a limitation in our study and I have indicated that.
- Regarding barriers and facilitators, the authors seem to treat Africa as a monolithic entity when discussing challenges like political unrest or climate conditions. Given that the study encompasses multiple countries in Africa, it would be beneficial to include a comparative analysis. Specifics on how different environmental or political conditions affected CHW programs in each country would give a nuanced view and avoid over-generalizations. Could you specify which countries faced what kinds of challenges to provide a more comprehensive understanding?
- RESPONSE: Thank you for your comment. We absolutely agree that the multiple countries in Africa are unique and have different environmental or political conditions that impacted CHW implementation. For that reason we created a detailed supplement (S1.Figure) that has the extracted barriers and facilitators outlining exactly the study and country details in relation to political, cultural, and environmental barriers/facilitators. We provide an overview in the manuscript and refer to the figure to which summarizes the details extracted for each project. This has been clarified in the manuscript as well (lines 301-350).
